# ZNF9 Activation of IRES-Mediated Translation of the Human ODC mRNA Is Decreased in Myotonic Dystrophy Type 2

**DOI:** 10.1371/journal.pone.0009301

**Published:** 2010-02-18

**Authors:** Morgan A. Sammons, Amanda K. Antons, Mourad Bendjennat, Bjarne Udd, Ralf Krahe, Andrew J. Link

**Affiliations:** 1 Department of Biological Sciences, Vanderbilt University, Nashville, Tennessee, United States of America; 2 Department of Medical Genetics, Folkhalsan Institute of Genetics, University of Helsinki, Finland; 3 Neuromuscular Research Unit, Tampere University Hospital and Medical School, Tampere, Finland; 4 Department of Neurology, Vasa Central Hospital, Vasa, Finland; 5 Department of Genetics, University of Texas M.D. Anderson Cancer Center, Houston, Texas, United States of America; 6 Department of Microbiology and Immunology, Vanderbilt University School of Medicine, Nashville, Tennessee, United States of America; Lehigh University, United States of America

## Abstract

Myotonic dystrophy types 1 and 2 (DM1 and DM2) are forms of muscular dystrophy that share similar clinical and molecular manifestations, such as myotonia, muscle weakness, cardiac anomalies, cataracts, and the presence of defined RNA-containing foci in muscle nuclei. DM2 is caused by an expansion of the tetranucleotide CCTG repeat within the first intron of *ZNF9*, although the mechanism by which the expanded nucleotide repeat causes the debilitating symptoms of DM2 is unclear. Conflicting studies have led to two models for the mechanisms leading to the problems associated with DM2. First, a gain-of-function disease model hypothesizes that the repeat expansions in the transcribed RNA do not directly affect ZNF9 function. Instead repeat-containing RNAs are thought to sequester proteins in the nucleus, causing misregulation of normal cellular processes. In the alternative model, the repeat expansions impair ZNF9 function and lead to a decrease in the level of translation. Here we examine the normal *in vivo* function of ZNF9. We report that ZNF9 associates with actively translating ribosomes and functions as an activator of cap-independent translation of the human ODC mRNA. This activity is mediated by direct binding of ZNF9 to the internal ribosome entry site sequence (IRES) within the 5′UTR of *ODC* mRNA. ZNF9 can activate IRES-mediated translation of ODC within primary human myoblasts, and this activity is reduced in myoblasts derived from a DM2 patient. These data identify ZNF9 as a regulator of cap-independent translation and indicate that ZNF9 activity may contribute mechanistically to the myotonic dystrophy type 2 phenotype.

## Introduction

Myotonic dystrophy types 1 and 2 are forms of muscular dystrophy resulting from large expansions of nucleotide repeats within noncoding regions of *DMPK* and *ZNF9*, respectively [1]. Myotonic dystrophy type 1 (DM1) is caused by expansion of a CTG triplet repeat within the 3′ untranslated region (UTR) of *DMPK*, whereas myotonic dystrophy type 2 (DM2) results from expansion of a CCTG repeat within the first intron of *ZNF9* [2], [3]. Despite the different expansions within two apparently unrelated genes, both diseases share many common clinical manifestations, including myotonia, muscle weakness, cataract development, insulin resistance, and cardiac conduction defects. The shared symptoms suggest the mechanisms causing the disease may also be shared [4], [5]. Although they have similar symptoms, there are also a number of very dissimilar features making them clearly separate diseases [6]. A major difference between DM1 and DM2 is that only DM1 shows a congenital form of the disorder [1]. Other apparent differences are the distribution of clinical muscle weakness/atrophy and the morphological involvement of different fiber types. In DM1, weakness and atrophy involves distal, facial, bulbar and respiratory muscles, whereas in DM2 the proximal muscles are preferentially involved, and the patients have marked muscle pains [7]. DM1 type 1 fibers show atrophy changes whereas in DM2 a subpopulation of type 2 fibers are highly atrophic [8]. These phenotypic differences suggest that other cellular and molecular pathways are involved besides the shared molecular pathomechanisms.

In both DM1 and DM2, the expanded nucleotide repeats are transcribed but are not translated. For *ZNF9*, the transcribed CCTG repeats within the first intron are apparently removed from both the wild-type and DM2 pre-mRNAs by splicing, resulting in translation of the normal ZNF9 protein [9]. Two studies of ZNF9 mRNA and protein expression levels reported no change in diseased myoblasts or lymphoblastoid cell lines compared to unaffected cells [9], [10]. These results indicate that the repeat RNA does not affect normal ZNF9 function and have led to a disease model in which the expanded repeats somehow dominantly interfere with other cellular processes [11], [12], [13], [14]. This model is supported by the observation that expression of transgenically engineered expanded CTG repeats in unrelated genes can cause some of the phenotypes seen in the myotonic dystrophies [15]. This toxic-RNA gain-of-function disease model hypothesizes that the transcribed CTG or CCTG repeat expansions fold into RNA structures that are retained in the nucleus forming distinct foci. Experimental data shows the ribonuclear complexes sequester similar sets of proteins in the nucleus, apparently causing misregulation of normal cellular processes, in particular RNA splicing [16], [17], [18]. Most prominently, the transcribed CTG and CCTG repeats both bind muscleblind-like (MBNL) and CUGBP1/ETR-3-like factors (CELF) that are involved in alternative RNA splicing.

In contrast to the toxic-RNA gain-of-function model, several studies have revealed a role for ZNF9 in DM2. Knockout of *ZNF9* in mice results in embryonic lethality due to defects in forebrain and craniofacial development [19]. Other studies in chick and zebrafish show that ZNF9 deletion results in severe brain and muscle phenotypes [20], [21]. Mice heterozygous for the *znf9* knockout display late-onset muscle wasting, cardiac abnormalities, cataracts, and mRNA expression defects similar to those seen in DM2 [22]. These defects can be rescued by reintroduction of wild-type levels of ZNF9, suggesting that a loss of ZNF9 function likely contributes to DM2. Similar to DM2, DMPK deficient mice show a subset of the phenotypes seen in DM1 patients [23]. A recent study reports that expanded CCTG repeats in DM2 give rise to defects in ZNF9 expression and cellular localization [24]. The discrepancy between this study and earlier characterizations of ZNF9 expression and localization in DM2 cells is unclear. This study reported that decreased ZNF9 activity is linked to a downregulation of translation [24]. A second study has reported that the expression of the CCTG repeats in mouse and human myoblasts alters translation and protein degradation [25]. These data support the notion that the ZNF9 protein may play a role in DM2 and that the RNA-mediated dominant gain-of-function model for myotonic dystrophy is not the only pathomechanism for the disease.

The lack of an *in vivo* biochemical function for ZNF9 has hindered the ability to clearly define the role that ZNF9 plays in DM2. ZNF9 has been proposed to act in a variety of cellular functions, including transcription, splicing, and translation [19], [26], [27], [28], [29]. Recent evidence suggests that ZNF9 likely acts as a regulator of translation. ZNF9 associates with 5′ terminal oligopyrimidine (TOP) mRNA elements [30], and 5′ TOP mRNAs are inefficiently translated in DM2 [24]. Previously, our group identified ZNF9 as a positive regulator of IRES-mediated translation for the rat ornithine decarboxylase (ODC) mRNA [26], providing further evidence that ZNF9 functions in translational regulation.

We hypothesized that ZNF9 acts as an IRES *trans*-activating factor (ITAF) for cap-independent translation of the human ODC mRNA. By analyzing the *in vivo* function of ZNF9 as an activator of cap-independent translation, we sought to determine whether a loss in ZNF9 activity contributes to DM2. The data presented here suggest that ZNF9 directly interacts with the IRES of the human ODC mRNA, associates with translating ribosomes, and activates cap-independent translation. Additionally, our data show that ZNF9 activity is reduced in myoblasts from a patient affected with DM2, providing further evidence that a loss-of-function mechanism contributes to myotonic dystrophy type 2 disease.

## Materials and Methods

### Cell Culture

HEK293T and HeLa cell lines were grown in DMEM containing 10% fetal bovine serum and penicillin/streptomycin solution according to standard laboratory practices (ATCC). Control and DM2 human myoblasts were grown in Complete Myoblast Medium (Promocell GmbH, Heidelberg, Germany) and cultured according to the manufacturer's recommendations. DM2 myoblasts were established from muscle biopsies from a male patient in his early 40's with clinical symptoms of DM2. The (CCTG)n repeat size was approximately 4500 repeats, as determined using established methods [31].

### Plasmid Construction

The full-length 5′UTR of the human ODC mRNA from clone #5088190 (Magic Consortium CloneID) was inserted between the *Renilla* and firefly luciferase genes to create pcDNA3.1-hODC-IRES. pcDNA3.1-V5-ZNF9 was constructed by insertion of the PCR-generated full-length open reading frame of human ZNF9 into pENTR and subsequent recombination into pcDNA3.1/nV5-Dest (Invitrogen) by recombinational cloning (Invitrogen). The PCR primers for cloning ZNF9 were 5′- CACCATGAGCAGCAATGAGTGCTT-3′ and 5′- TTATTAGGCTGTAGCCTCAATTGTGCA-3′. pcDNA3.1-V5-LacZ was created by recombinational cloning of pcDNA3.1/nV5-GW/*lac*Z into pcDNA3.1/nV5-Dest. An HIV –based reporter vector (hODC-IRES) was cloned by introduction of the full-length hODC-IRES coding sequence into H163 [32]. pcDNA3.1-hODC-IRES was digested with *Kpn*I and *Not*I, blunt-ended with Klenow fragment, and inserted into the *Sma*I site of H163. HIV-ZNF9-myc and HIV-LacZ-Myc were created by digestion of pcDNA3.1-ZNF9-myc or pcDNA3.1-LacZ-myc with *Kpn*I and *Pme*I. The fragments were blunt-ended with Klenow fragment and inserted into the *Sma*I site of H163. All clones were verified by DNA sequencing.

### Western Blotting

Rabbit polyclonal antibodies were generated against full-length, recombinant ZNF9 (Covance). Briefly, recombinant human ZNF9 was expressed in *E. coli* (BL21DE3) as an N-terminal GST fusion protein and purified by glutathione affinity chromatography. ZNF9 without the GST tag was released from the column by cleavage with 3C protease (Prescission Protease, GE). The purity and identity of ZNF9 were determined by Coomassie staining and mass spectrometry analysis. ZNF9 antibodies were affinity purified against full-length recombinant ZNF9. Antibodies for immunoblotting were used at the following concentrations: ZNF9 (1∶1,000), GAPDH (Millipore) (1∶5,000), c-myc (Cell Signaling) (1∶1,000), anti-myc epitope (Vanderbilt Monoclonal Antibody Core) (1∶2,000), V5 (Invitrogen) (1∶2,500).

### Subcellular Fractionation

HeLa cells were transfected with either pcDNA3.1-ZNF9-myc or pcDNA3.1-LacZ-myc using Lipofectamine 2000 (Invitrogen) according to the manufacturer's recommendations. Transfected cells were fractionated into whole cell, cytoplasmic, and nuclear fractions using the NE-PER Nuclear and Cytoplasmic Extraction reagents (Thermo Scientific) according to the manufacturer's recommendations.

### Quantiative RT-PCR

Total RNA was isolated from cultured cells using TRIzol according to manufacturer's recommendations (Invitrogen). RNA was quantified using a Nanodrop spectrophotometer (Thermo Scientific). 1 µg of RNA was DNase-treated (Promega) and used in reverse transcription reactions primed with random hexamers (Superscript III, Invitrogen). cDNA samples were treated with RNase H and 5 µL of each sample were used for quantitative real-time PCR reactions. Real-time PCR was performed using the Applied Biosystems 7000 and the manufacturer's protocols (Applied Biosystems). Taqman probes used were: ZNF9 (Hs00231535_m1) and GAPDH (4333764T).

### Sucrose Gradient Analysis and Centrifugation

Polysome analysis was performed as previously described [26]. Intact cells were lysed by repeated vortexing in five pellet volumes of lysis buffer (100 mM Tris, 10 mM MgCl_2_, 100 mM KCl, and 1% TritonX-100), and cellular debris was removed by centrifugation (4°C, 13,500x*g*). 10 O.D. units of lysate were loaded onto a 7–47% sucrose gradient. Unless indicated, sucrose solutions contained 10 mM Tris-HCl (pH 7.5), 5% cycloheximide, 5 mM MgCl_2_, and 100 mM EDTA. Gradients were centrifuged for 2 h at 178,000x*g* in a Sorvall SW-41 swinging bucket rotor. Polysome analysis was performed by bottom displacement and monitored by UV absorbance at 254 nm. Fractions were collected for western blotting. Ribosome pelleting experiments were performed by centrifugation of the cell lysates described above through a 1 M sucrose cushion (−/+50 mM EDTA) at 100,000x*g* for 2 h in a TLA120.2 rotor. The resulting ribosome pellets were resuspended in ribosome solubilization buffer (20 mM TRIS pH 7.5, 5 mM MgCl_2_, 6 M urea). Supernatant fractions were precipitated with trichloroacetic acid, washed with acetone, and resuspended in ribosome solubilization buffer. For ribosome salt wash analysis, ribosome pellets were isolated as described above, and the supernatants were discarded. Pellets were washed in ice cold PBS and resuspended in ribosome salt wash buffer containing 20 mM Tris-HCl (pH 7.5), 5 mM MgCl_2_, 1 mM BME, and either 100 mM, 250 mM, 500 mM, or 1 M potassium acetate. Resuspended ribosomes were then centrifuged at 100,000x*g* for 2 h in a TLA120.2 rotor. Supernatant fractions were removed and TCA precipitated as described above, and pellet fractions were resuspended in solubilization buffer. Equal volumes of lysate were separated by polyacrylamide gel electrophoresis, transferred to nitrocellulose membranes, and immunoblotted for ZNF9 protein.

### *In Vitro* RNA Synthesis and Binding Assays

RNA probes were synthesized using double-stranded DNA templates with upstream T7 promoters using Ambion's MegaShortScript kit according to the manufacturer's recommendations. The DNA primers used were: 5′-TAA TAC GAC TCA CTA TAG G-3′ and 5′-GAT TTC TTG ATG TTC CTA TGG AAA ACT AAG AGA TGG AAT TGA AAG AAA CCT ATA GTG AGT CGT ATT A-3′. The sequence of the synthesized RNA probe is 5′-UUUCUUUCAAUUCCAUCUCUUAGUUUUCCAUAGGAACAUCAAGAAAUC-3′. The RNA probe was purified by TRIzol extraction (Invitrogen), and the size and purity were determined by gel electrophoresis. Probes were end labeled with ^32^P using T4 polynucleotide kinase according to the manufacturer's recommendations (NEB). All RNA binding reactions were performed with 20 pmol of labeled RNA in a reaction volume of 30 µL of RNA binding buffer (150 mM NaCl, 100 mM NaHPO_4_, and 5% glycerol) in a clear-bottom 96 well plate at room temperature, unless otherwise indicated. UV cross-linking reactions were performed on a 302 nm UV lightbox for 5 min at room temperature. Reactions were stopped by addition of Laemmli buffer. The reactions were analyzed by polyacrylamide gel electrophoresis and transferred to nitrocellulose membranes. Membranes were stained with Ponceau S to measure ZNF9 protein levels, and exposed to film for imaging of the radioactive signals.

### Luciferase Reporter Assays

HeLa cells were grown in 24-well plates to 70% confluence and transfected with the human ODC-IRES reporter and either pcDNA3.1-V5-LacZ or pcDNA3.1-V5-ZNF9 using Lipofectamine 2000 according to manufacturer's recommendations (Invitrogen). Cells were harvested after 24 h, and dual luciferase reporter assays were performed according to the manufacturer's recommendations (Promega) using a Turner TD-20/20 lumninometer.

### Mass Spectrometry-Based Proteomics

Sample preparation and mass spectrometry-based proteomics analysis were performed as described earlier [26], [33].

### Generation of shRNA Virus

Double-stranded DNA sequences were generated and cloned into pENTR/U6 using the Block-iT U6 RNAi Entry vector kit (Invitrogen). Primer sequences were ZNF9-198 Top strand 5′- CAC CGC AGC AAT GAG TGC TTC AAG TAG AGC TTG ACT TGA AGC ACT CAT TGC TGC -3′, ZNF9-198 Bottom strand 5′-AAA AGC AGC AAT GAG TGC TTC AAG TCA AGC TCT ACT TGA AGC ACT CAT TGC TGC -3′, ZNF9-645 Top Strand 5′-CAC CGC AAG ACA AGT GAA GTC AAC TAG AGC TTG AGT TGA CTT CAC TTG TCT TGC -3′, ZNF9-645 Bottom Strand 5′-AAA AGC AAG ACA AGT GAA GTC AAC TCA AGC TCT AGT TGA CTT CAC TTG TCT TGC -3′. Ligation products were transformed into One Shot TOP10 competent *E. coli* (Invitrogen). Minipreps were performed on selected colonies and shRNA fidelity was verified by DNA sequencing. Gateway recombination reactions were performed by transfering the shRNA constructs into the lentiviral vector system as described previously [34]. Lentiviruses encoding ZNF9 or control shRNAs were generated by calcium-phosphate mediated co-transfection of HEK293T cells with the resulting shRNA-containing lentiviral vector, VSV-G, and pol/gag. VSV-G and pol/gag vectors were kind gifts of Dr. D. Unutmaz, NYU School of Medicine [35]. The resulting viral supernatants were concentrated using Amicon 100K cut-off concentrators (Millipore).

### Viral Infection and Knockdown of ZNF9

Viral supernatants were applied to 1×10^6^ HeLa cells cultured under standard conditions. Infections were performed at an MOI of 2 and were allowed to proceed for 48 h. GFP+ cells were sorted and collected using a FACSAria flow sorter (BD Biosciences) and placed back in culture. Knockdown of target genes was assessed by quantitative RT-PCR (Taqman) and western blotting directed at the target protein.

### Infection of Primary Myoblasts

HIV-hODC-IRES reporter, HIV-ZNF9-myc, and HIV-LacZ-myc viral supernatants were generated by calcium phosphate mediated co-transfection of the HIV vector and pL-VSV-G into HEK293T cells cultured in DMEM with 10% FBS [32]. The medium was changed after 6 h and replaced with myoblast complete growth medium. Supernatants were collected 48 h after transfection and were sterile filtered and stored at −80°C. Primary myoblasts were seeded at 15,000 cells per well in myoblast complete growth medium. Viral supernatants were added to the cells for 8 h, the medium was changed to standard growth medium, and the cells were subsequently assayed after 48 h for luciferase activity as described above.

### Statistical Methods

Two-tailed Student's T-tests (95% confidence interval) were performed on pairwise combinations of data to determine statistical significance.

## Results

### ZNF9 Specifically Binds to a Cellular mRNA IRES Element

**Figure 1:**
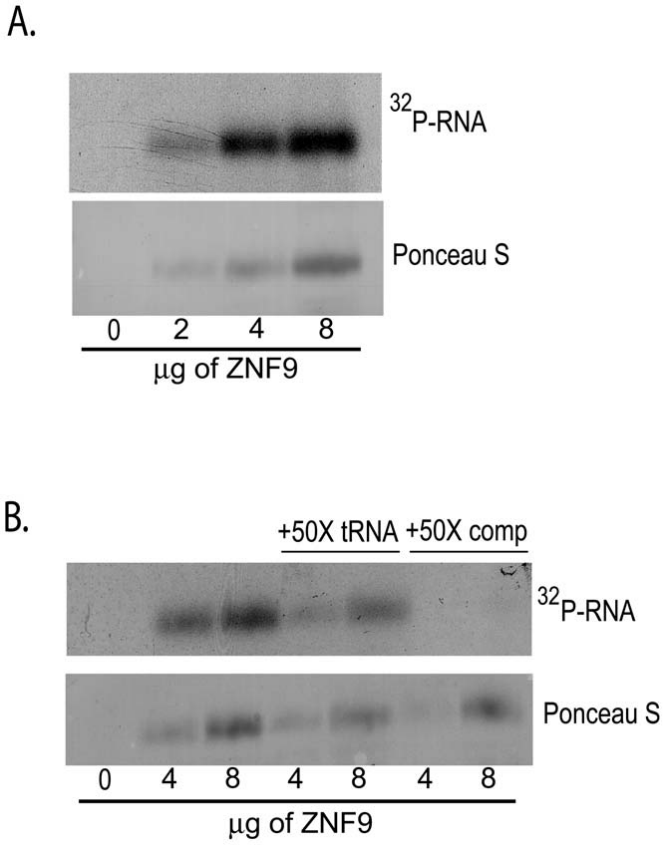
ZNF9 specifically binds to a human cellular mRNA IRES element. A. Increasing amounts of recombinant, full-length ZNF9 were incubated with ^32^P-end-labeled ODC RNA probe and cross-linked. Reactions were separated by polyacrylamide gel electrophoresis, transferred to nitrocellulose and exposed to X-ray film. Membranes were stained for ZNF9 protein using Ponceau S. B. Recombinant ZNF9 was incubated with ^32^P-end-labeled ODC RNA probe and either a 50-fold molar excess of bovine liver tRNA or a 50-fold molar excess of unlabeled ODC RNA competitor (comp) probe. Samples were processed as in *A*.

Previously, we identified ZNF9 in a screen for proteins binding to an IRES element from the rat ornithine decarboxylase (ODC) mRNA [26], [36]. It was unknown whether ZNF9 directly binds the ODC's IRES RNA sequence or if it binds in the presence of other protein cofactors. We tested whether ZNF9 can bind directly to a 45 bp region in the human ODC mRNA corresponding to the rat IRES-containing sequence. In Figure 1A, we show that ZNF9 directly binds to the 45 bp human ODC IRES RNA sequence. The interaction is sequence specific since the interaction was abolished with the addition of a 50-fold molar excess of an unlabeled competitor identical to the probe (Fig. 1B). In contrast, the addition of a 50-fold molar excess of a non-specific competitor RNA did not reduce or eliminate ZNF9 binding to the ODC IRES RNA probe (Fig. 1B). The ability of ZNF9 to bind to a putative IRES element in the absence of other proteins suggests that it may act as an ITAF through direct recognition of the IRES sequence. One possible mechanism for ITAF function involves ZNF9 acting as a physical scaffold between an IRES-containing mRNA and the ribosomal machinery. Tethering of the mRNA to the ribosome by an ITAF is thought to facilitate translation of the mRNA [37], [38], [39]. In order to test this model for ZNF9, we investigated the interactions of ZNF9 with the translation machinery.

### ZNF9 Interacts with the Translating Eukaryotic Ribosome

**Figure 2:**
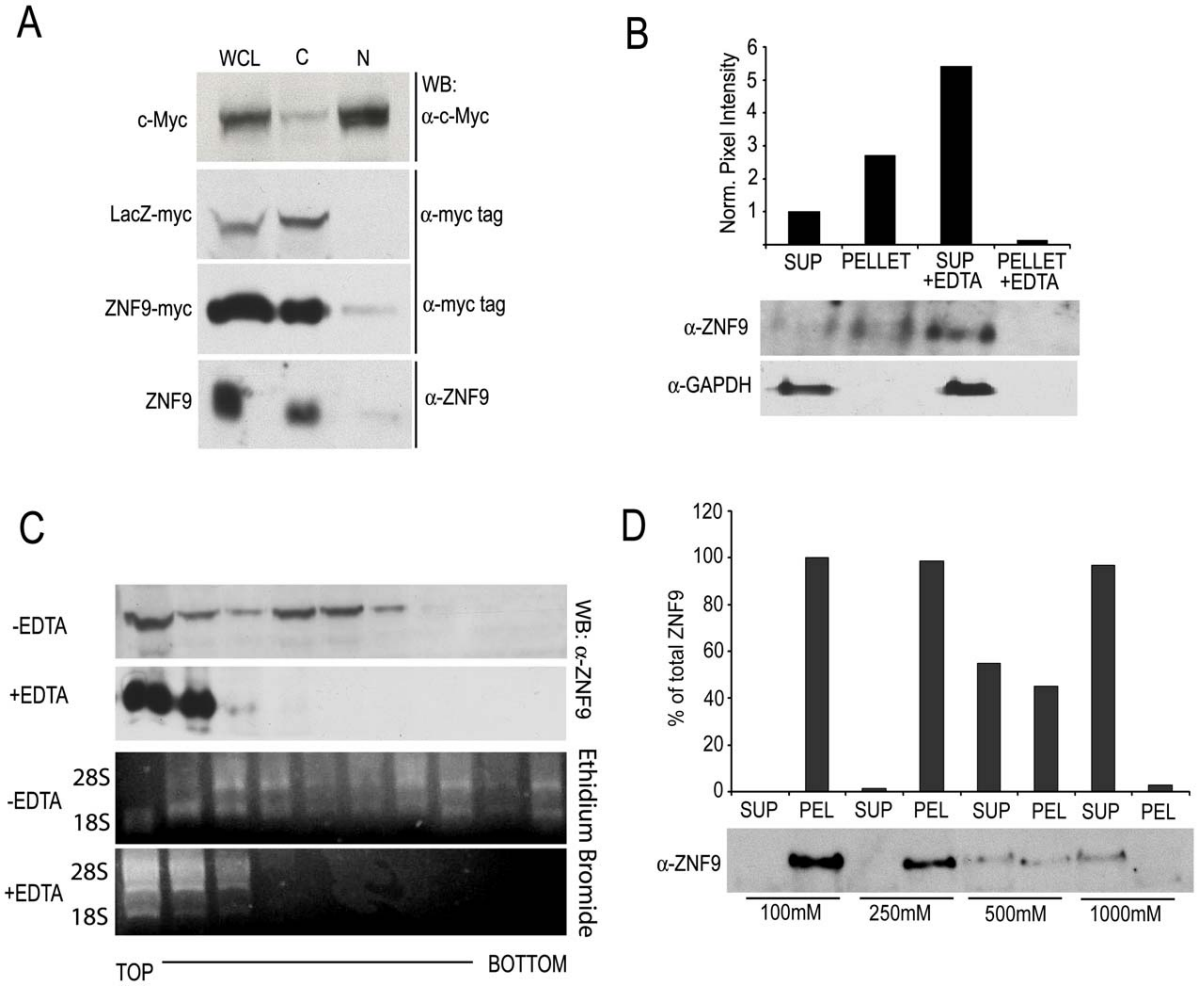
ZNF9 interacts with the translating human eukaryotic ribosome. A. Western blots of sucrose gradient fractions of HeLa cells extracts transfected with either ZNF9-myc or *LacZ*-myc and probed with the indicated antibodies. 10% of each fraction was analyzed by western blotting. WCL: whole cell extract, C: cytoplasmic, N: nuclear B. ZNF9 and GAPDH western blotting of supernatant and polysome fractions of HeLa cell lysates isolated in the absence or presence of EDTA. Quantification was normalized to the levels of ZNF9 in the supernatant fraction in the absence of EDTA C. ZNF9 western blotting of sucrose gradient fractions of HeLa cell lysates in the absence or presence of EDTA. Equal cell lysate amounts (10 O.D. units) were applied to each gradient (−/+50 mM EDTA), and each fraction was TCA precipitated. 50% of the total precipitated protein was analyzed by western blotting. D. ZNF9 western blotting analysis of supernatant and pellet fractions from ribosome salt wash experiments. Western blots were quantified and the data are reported as ratios of ZNF9 in the pellet fraction to the amount of ZNF9 in the supernatant, normalized to the values for 100 mM potassium acetate.

Based on its proposed role as an ITAF for cap-independent translation, we hypothesized that ZNF9 interacts with translating ribosomes. Previous work from our lab showed that ZNF9 co-sediments with the polyribosome fractions, suggesting an interaction with the ribosome and a role in translation [26]. Therefore, we sought to analyze the intracellular localization of ZNF9 and whether ZNF9 associates with the ribosome during active translation. Subcellular fractionation experiments revealed that the majority of ZNF9 protein is found in the cytoplasm (Fig. 2A). Both myc-tagged and endogenous ZNF9 localize to the cytoplasm (Fig. 2A). Western blotting for a known nuclear protein (c-myc) and a cytoplasmic protein (β-galactosidase) were used as controls for the selectivity of the fractionation technique. Both control proteins were found exclusively in the expected fraction. A small amount (∼5%) of the total ZNF9 found in the cell was localized to the nucleus. While this may be carryover from the cytoplasmic fraction, the experimental and control results suggest that a small fraction of ZNF9 is nuclear. The significance and function of nuclear ZNF9 are unknown, but ZNF9 has been shown previously to have a nuclear pool [24], [25]. The cytoplasmic localization of ZNF9 is consistent with its proposed role in translation.

Differential centrifugation of high molecular weight complexes is a well-established technique for the isolation of polyribosomes [40]. To determine if ZNF9 copurifies with human polysomes, lysates from HeLa cells were loaded onto a sucrose cushion and the polyribosome fraction was isolated. The majority of ZNF9 is found in the pellet (polyribosome) fraction, which suggests that ZNF9 associates with translating ribosomes (Fig. 2B). A fraction of ZNF9 protein remains in the supernatant, suggesting that a portion of ZNF9 protein is not actively engaged with the ribosome (Fig. 2B). As a control, GAPDH, a protein that does not interact with translating ribosomes, is absent from the pellet fraction and is exclusively localized to the supernatant. The addition of EDTA disrupts polyribosome formation and is often used as a control to rule out association with other high molecular weight complexes that co-sediment with ribosomes [33]. In the presence of EDTA, ZNF9 is found exclusively in the supernatant fraction (Fig. 2B), suggesting that the ZNF9 sedimentation in the absence of EDTA is due to an association with the ribosome and not a separate protein complex. We also analyzed ZNF9 sedimentation with ribosomes by linear sucrose gradient ultracentrifugation and confirmed that ZNF9 associates with very high molecular weight particles and that this interaction is abolished by the addition of EDTA (Fig. 2C). As seen previously, a fraction of ZNF9 does not sediment in the ribosome-containing fractions, suggesting that a pool of ZNF9 is not actively engaged in translation.

In order to further test our hypothesis that ZNF9 acts as a translation initiation factor for cap-independent translation, we sought to test how tightly ZNF9 is associated with ribosomal proteins. Ribosome salt wash experiments are often used to separate core ribosomal protein subunits from associated translation factors [41]. The assay is also used to estimate the relative strength of protein-ribosome interactions by increasing the salt concentration [42]. We hypothesized that, like other translation factors, ZNF9 would be released from the polysomes with intermediate concentrations of salt. At low concentrations of potassium acetate, ZNF9 localizes exclusively to the ribosome pellet (Fig. 2D). At 500 mM potassium acetate, a concentration that is sufficient to strip most translation factors from ribosomes [40], ZNF9 is partially dissociated from the ribosome. At 1 M potassium acetate, ZNF9 protein is completely removed from the ribosome, suggesting that ZNF9-ribosome interactions are not as strong as the core ribosomal subunits. In summary, our data indicate that ZNF9 associates with translating polysomes and that the strength and mode of the interaction is consistent with those of other translation factors [33], [40].

### ZNF9 Activates Cap-Independent Translation of the Human ODC mRNA

**Figure 3:**
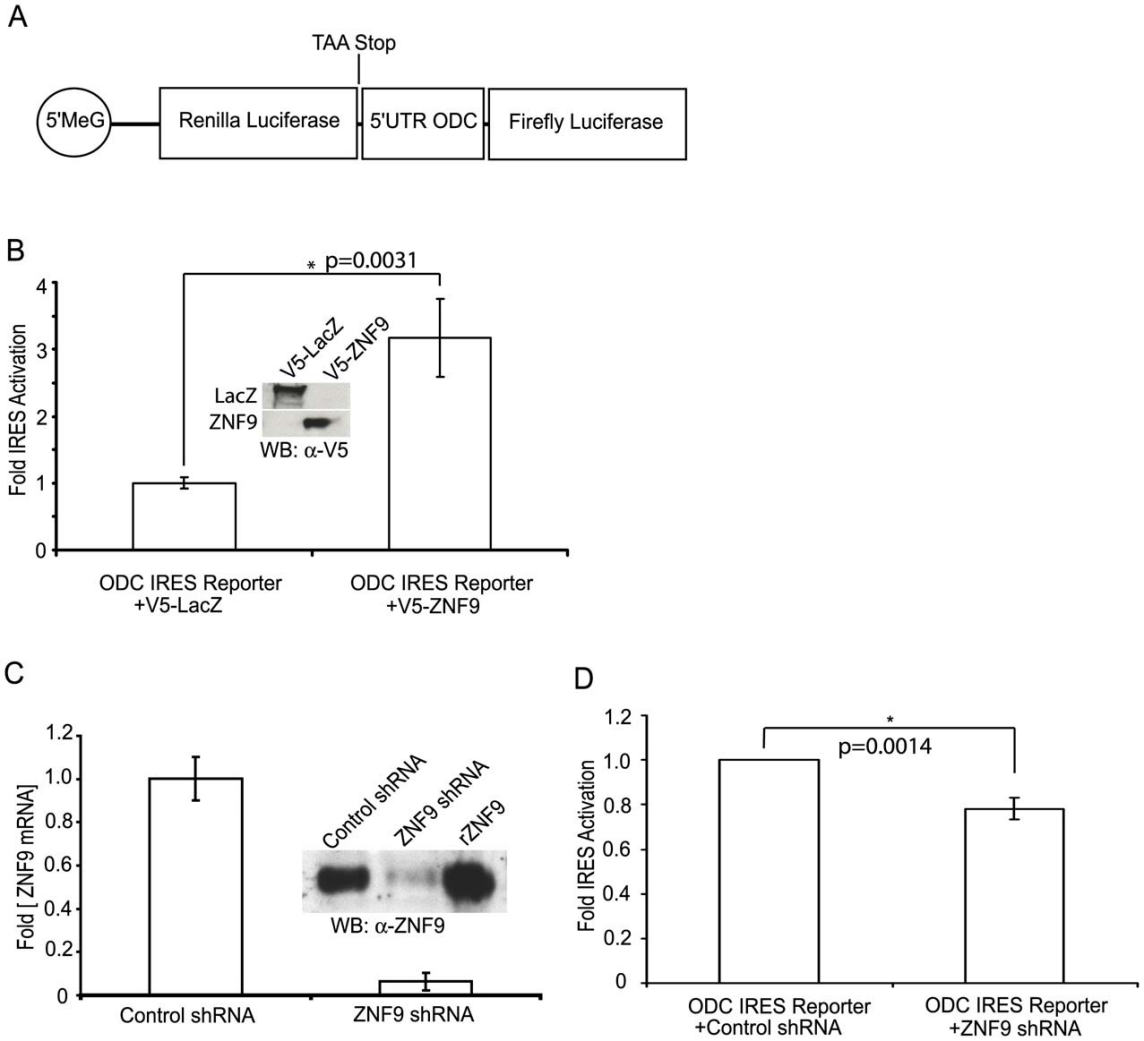
ZNF9 activates cap independent-translation of the human ODC mRNA. A. Diagram of the ODC bicistronic vector for measuring cap-independent translation of human ODC. 5′meG: 5′-methyl-G cap structure. B. HeLa cells expressing the ODC bicistronic vector and either V5-ZNF9 or V5-LacZ were analyzed for luciferase activity. The level of cap-independent translation is reported as a ratio of cap-independent translation (firefly) to cap-dependent translation (*Renilla*). Values are averages of 6 independent experiments. The error bars are the standard error of the mean (S.E.M). The insert figure is a representative western blot analysis showing the V5-LacZ and V5-ZNF9 levels in the HeLa cells lysates. C. ZNF9 mRNA and protein levels after RNAi of ZNF9 in HeLa cells. HeLa cells expressing either a control shRNA or a ZNF9-specific shRNA were analyzed using quantitative RT-PCR and western blotting to quantify expression of ZNF9 RNA and protein, respectively. The RT-PCR values were computed using the ΔΔCt method and are normalized to the levels of GAPDH mRNA. Error bars represent the standard error. The insert figure is a western blot showing the ZNF9 proteins levels. Recombinant ZNF9 (rZNF9) was used as a positive control, and equal amounts of protein were loaded in each lane as assessed by BCA protein assays. D. HeLa cells expressing the ODC bicistronic vector and either a control or ZNF9-specific shRNA were assessed for luciferase activity. The level of cap-independent translation is reported as a ratio of cap-independent translation (firefly) to cap-dependent translation (*Renilla*). Values are the averages of 3 independent experiments and are normalized to the control shRNA. The error bars are the S.E.M.

We have determined that ZNF9 binds specifically to the 5′UTR of the human ODC mRNA and interacts with translating ribosomes. Previous data from our lab revealed that ZNF9 can activate cap-independent translation of the rat ODC mRNA, which is similar, but not identical, to the human ODC mRNA. These data suggest a direct role for ZNF9 in regulating translation of the ODC mRNA. To test if ZNF9 promotes human ODC IRES activity, a bicistronic reporter plasmid with the full-length human ODC 5′UTR was used to test the ability of ZNF9 to activate cap-independent translation (Fig. 3A). Overexpression of ZNF9 in HeLa cells resulted in an ∼3-fold increase (p-value = 0.0031) in translation of the second cistron of the reporter compared to control experiments with β-galactosidase overexpression (Fig. 3B). These data, along with a demonstration of direct ZNF9 binding to the 5′UTR of ODC, strongly suggests that ZNF9 acts as a translation factor for cap-independent translation of the human ODC mRNA.

In order to validate the role for ZNF9 in cap-independent translation of the human ODC mRNA, we designed specific short hairpin RNA constructs (shRNAs) to knockdown expression of ZNF9 mRNA. The short hairpin constructs were expressed in a lentiviral system for infection into HeLa cells, and infected cells were selected for stable GFP expression by flow cytometry. Cells expressing the ZNF9-specific shRNA showed ∼90% reductions (p-value = 2.2×10^−5^) in ZNF9 mRNA levels and a reduction in ZNF9 protein levels compared to control shRNA-expressing cells, as assessed by quantitative RT-PCR and western blotting (Fig. 3C and insert). These cells with dramatically reduced levels of ZNF9 have ∼30% reduction (p-value = 0.0014) in cap-independent translation of ODC (Fig. 3D), suggesting that ZNF9 is required for full activation of cap-independent translation of the ODC mRNA.

### Cap-Independent Translation of ODC Is Defective in Human DM2 Myoblasts

**Figure 4:**
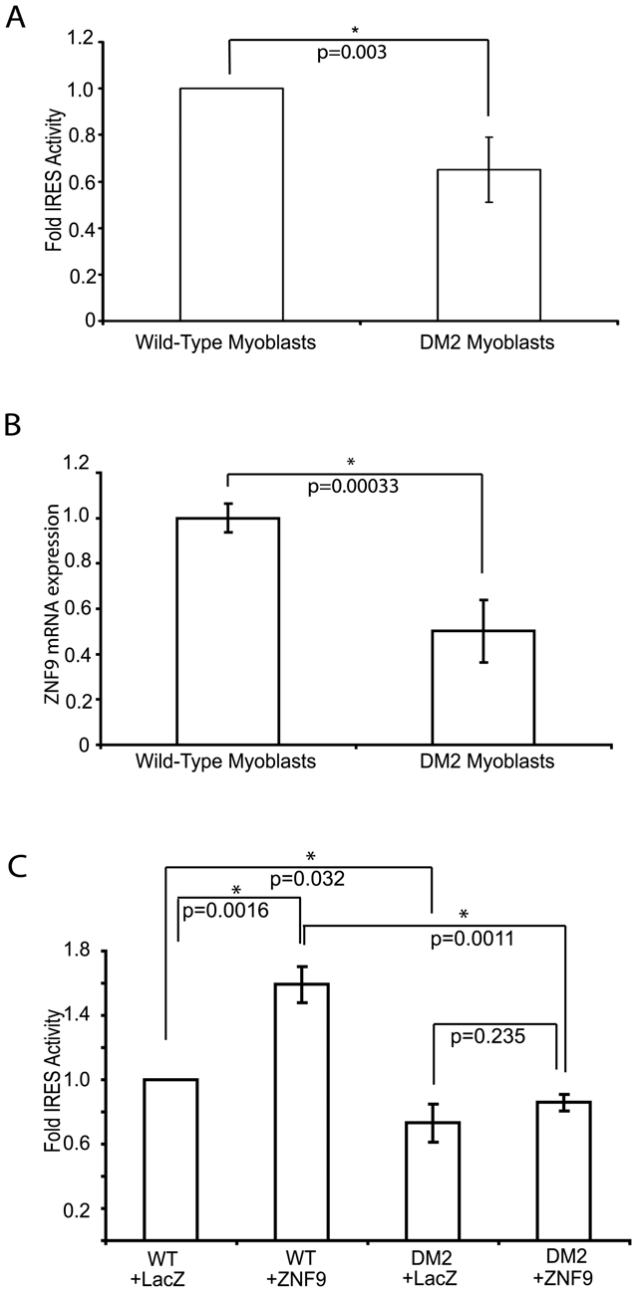
Cap-independent translation of ODC is defective in DM2 myoblasts. A. IRES activity in wild-type and DM2 myoblast. Equal numbers of human wild-type or DM2 myoblasts were infected with virions expressing the bicistronic ODC reporter vector and were analyzed for luciferase activity. The level of cap-independent translation is reported as a ratio of cap-independent translation (firefly) to cap-dependent translation (*Renilla*). Values are averages of 6 independent experiments and are normalized to IRES activity in wild-type myoblasts. B. Differences in ZNF9 mRNA expression in wild-type and DM2 myoblasts. Quantitative real-time PCR was performed on equal amounts of total mRNA from wild-type and DM2 myoblasts. Values were computed using the ΔΔCt method and are normalized to levels of GAPDH mRNA. Error bars represent the S.E.M. C. IRES activity in wild-type and DM2 myoblast overexpressing ZNF9. Equal numbers of wild-type or DM2 myoblasts were infected with the bicistronic ODC reporter vector and vectors for either the overexpression of LacZ or ZNF9. Cell lysates were analyzed for luciferase activity as described above. Values are reported as a ratio of cap-independent translation to cap-dependent translation and are representative of 4 independent experiments. The values are normalized to the value of IRES activation in wild-type myoblasts overexpressing LacZ. Error bars represent the S.E.M.

Our data show that ZNF9 stimulates the cap-independent translation of the ODC mRNA. Using a biochemical assay for ZNF9 function, we sought to address the critical question of whether the repeat expansions that cause human DM2 also compromise this function of ZNF9. If ZNF9 activity is reduced in DM2, we predicted that translation of the ODC mRNA would be reduced compared to wild-type myoblasts. A bicistronic reporter (HIV-hODC) for cap-independent translation of ODC was introduced into normal and primary human DM2 myoblasts by viral infection. We found cap-independent activation of ODC function is reduced in DM2 (Fig. 4A). DM2 myoblasts show a 31% reduction (p-value = 0.003) in cap-independent translation of the ODC reporter (Fig. 4A), providing evidence that cap-independent translation is reduced in DM2. Using quantitative RT-PCR for ZNF9, we determined that the DM2 myoblasts used in our analysis have ∼50% of the ZNF9 mRNA (p-value = 0.0003) found in wild-type myoblasts (Fig. 4B), similar to what was observed in an earlier study [24].

To test if the reduction in cap-independent translation results from loss of ZNF9 activity, we tested whether overexpression of ZNF9 would rescue the defect. We overexpressed ZNF9 or β-galactosidase in wild-type and DM2 myoblasts by infection with HIV-based virions. Overexpression of ZNF9 in wild-type myoblasts increased cap-independent translation 59% (p-value = 0.0016) as seen previously in the HeLa cell lines (Fig. 3B). Expression of β-galactosidase (*LacZ*) in WT and DM2 myoblasts had no discernible effect on cap-independent translation, as expected from the results shown in Figure 4A. Overexpression of ZNF9 in DM2 myoblasts results in a 12% increase (p-value = 0.235) in cap-independent translation of the ODC reporter compared to DM2 myoblast controls (Fig. 4C). The level of cap-independent translation in ZNF9-overexpressing DM2 myoblasts is not restored to the level of wild-type myoblasts (Fig. 4C). The results show that ZNF9 overexpression increases cap-independent translation in both wild-type and DM2 myoblasts, but ZNF9 overxpression in DM2 myoblasts is not sufficient to restore cap-independent translation of the ODC reporter to the level seen in wild-type myoblasts.

In summary, our experiments indicate that ZNF9 associates with actively translating polysomes and activates cap-independent translation of ODC in cultured cell lines. ZNF9 activates cap-independent translation in primary human myoblasts, and this activity is reduced in myoblasts from a DM2 patient. Overexpression of ZNF9 partially restores cap-independent translation in human DM2 myoblasts (Fig. 4C), suggesting that the repeat expansions causing DM2 probably contribute to the reduction of at least one aspect of ZNF9 function. However, other modes of regulating gene expression may be involved and cannot be ruled out.

## Discussion

Misregulation of translational control is a common cause of multiple diseases, including many cancers, fragile X syndrome, and vanishing white matter disease [43], [44], [45], [46]. Defects in cap-independent translation have been shown previously to be linked to diseases such as X-linked dyskeratosis congenita [47], suggesting that defects specifically in cap-independent translation can result in human diseases. Previous investigations into ZNF9 activity showed that reduced ZNF9 protein expression results in diminished translational activity and leads to symptoms that mimic those seen in myotonic dystrophy type 2 [22], [24], [25].

Our results support a model in which ZNF9 acts as an IRES trans-activating factor (ITAF) for cap-independent translation of ODC. ZNF9 binds directly to the 5′UTR of the ODC mRNA. ZNF9 is localized mainly to the cytoplasm and physically associates with actively translating ribosomes. Overexpression of ZNF9 activates cap-independent translation of a reporter construct in tissue culture cells. This ITAF activity is reduced in primary myoblasts from a myotonic dystrophy type 2 patient. These results are consistent with a model in which ZNF9 activity is reduced in DM2 patients.

Because ZNF9 interacts with both an mRNA and ribosomes, one likely mechanism for its ITAF activity is recognizing specific IRES sequences or structures within the 5′UTR of mRNAs and subsequently recruiting other initiation factors and the ribosome for translation activation [37], [38], [39]. It is unknown whether ZNF9 directly recruits the translation machinery or whether other factors are required. Both PCBP2 and La, known ITAFs, have been previously shown to interact with ZNF9 and similar RNA substrates [26], [28], [29], [48]. Interestingly, ZNF9 was found to interact with an assortment of single-strand RNA binding proteins and ribosomal protein subunits in a large-scale proteomic screen, consistent with the possibility that ZNF9 acts in concert with other proteins to activate translation [49]. Further examination of the interactions of ZNF9 with mRNAs and specific translation proteins will be important in elucidating the details of ribosome recruitment during cap-independent translation, currently a poorly understood process.

The two conflicting models for the cause of DM2 symptoms stem in part from conflicting data regarding the expression of ZNF9 mRNA and protein. Two separate studies noted no change in ZNF9 mRNA and protein levels in DM2 myoblasts or lymphoblastoid cell lines [9], [10]. These results support the prevailing toxic-RNA gain-of-function disease model in which the repeat expansions do not reduce ZNF9 activity but rather interfere with other critical processes. In contrast, Huichalaf *et al.* used both western blotting and *in situ* antibody staining in DM2 myoblasts to detect a decrease in ZNF9 protein levels that correlated with decreased translational activity [24]. These results are consistent with the loss-of-function disease model, in which the repeat expansions compromise ZNF9 function. The discrepancies may be due to low sample numbers, the use of unaffected tissue types (lymphoblastoid), or differences in the cell line's genetic backgrounds. A larger and more comprehensive analysis of ZNF9 protein levels in DM2 is warranted to resolve the discrepancies.

Our study of ZNF9 overexpression in DM2 myoblasts suggests that neither of the two models completely describes the situation in DM2. First, we find cap-independent translation is reduced but not eliminated in myoblasts from DM2 patients compared to myoblasts from healthy controls, a result expected from loss of ZNF9 function. The residual activity in DM2 myoblasts may stem from either low levels of ZNF9, partially active ZNF9, or ZNF9-independent activity. Overexpression of ZNF9 in normal myoblasts results in an increase in cap-independent translation. Thus, it seems reasonable to expect that overexpression of ZNF9 in DM2 myoblasts would restore cap-independent translation to at least normal levels. Cap-independent translation activity was slightly increased compared to control DM2 myoblasts and did not restore wild-type levels. This result suggests that activation of cap-independent translation by ZNF9 is somehow blocked in DM2 cells, possibly through a dominant-negative action of the expanded repeat RNA. Cell-free, reconstituted system in which ZNF9 levels can be carefully controlled will be important for a clearer understanding of this phenomenon.

Our data presented here and previous observations suggest that a combination of an RNA-mediated dominant effect and ZNF9 loss-of-function contribute to DM2. Salisbury *et al.* have shown that transcribed CCTG repeats are found in both the nucleus and the cytoplasm of DM2 myoblasts and that the RNA repeats misregulate translation [24], [25]. ZNF9 may be inhibited by the presence of transcribed CCTG repeats in the cytoplasm, much as the RNA splicing factor MBNL activity is thought to be inhibited by the presence of transcribed CTG and CCTG RNA in the nucleus [17], [50], [51]. Both proteins contain multiple zinc finger motifs to mediate binding to specific RNAs and may be inhibited by similar substrates. In future work, it will be important to determine whether ZNF9 and other RNA binding proteins are inhibited by the presence of transcribed CCTG repeats in either the nucleus or the cytoplasm.

## References

[1] Cho DH, Tapscott SJ (2007). Myotonic dystrophy: emerging mechanisms for DM1 and DM2.. Biochimica et Biophysica Acta.

[2] Aslanidis C, Jansen G, Amemiya C, Shutler G, Mahadevan M (1992). Cloning of the essential myotonic dystrophy region and mapping of the putative defect.. Nature.

[3] Liquori CL, Ricker K, Moseley ML, Jacobsen JF, Kress W (2001). Myotonic dystrophy type 2 caused by a CCTG expansion in intron 1 of ZNF9.. Science.

[4] Ranum LP, Day JW (2002). Myotonic dystrophy: clinical and molecular parallels between myotonic dystrophy type 1 and type 2.. Curr Neurol Neurosci Rep.

[5] Day JW, Ricker K, Jacobsen JF, Rasmussen LJ, Dick KA (2003). Myotonic dystrophy type 2: molecular, diagnostic and clinical spectrum.. Neurology.

[6] Udd B, Meola G, Krahe R, Thornton C, Ranum LP (2006). 140th ENMC International Workshop: Myotonic Dystrophy DM2/PROMM and other myotonic dystrophies with guidelines on management.. Neuromuscul Disord.

[7] Ricker K, Koch MC, Lehmann-Horn F, Pongratz D, Otto M (1994). Proximal myotonic myopathy: a new dominant disorder with myotonia, muscle weakness, and cataracts.. Neurology.

[8] Vihola A, Bassez G, Meola G, Zhang S, Haapasalo H (2003). Histopathological differences of myotonic dystrophy type 1 (DM1) and PROMM/DM2.. Neurology.

[9] Margolis JM, Schoser BG, Moseley ML, Day JW, Ranum LPW (2006). DM2 intronic expansions: evidence for CCUG accumulation without flanking sequence or effects on ZNF9 mRNA processing or protein expression.. Hum Mol Genet.

[10] Botta A, Caldarola S, Vallo L, Bonifazi E, Fruci D (2006). Effect of the [CCTG]n repeat expansion on ZNF9 expression in myotonic dystrophy type II (DM2).. Biochimica et Biophysica Acta (BBA) - Molecular Basis of Disease.

[11] Amack JD, Paguio AP, Mahadevan MS (1999). Cis and trans effects of the myotonic dystrophy (DM) mutation in a cell culture model.. Hum Mol Genet.

[12] Amack JD, Reagan SR, Mahadevan MS (2002). Mutant DMPK 3′UTR transcripts disrupt C2C12 myogenic differentiation by compromising MyoD.. The Journal of Cell Biology.

[13] Mankodi A, Takahashi MP, Jiang H, Beck CL, Bowers WJ (2002). Expanded CUG Repeats Trigger Aberrant Splicing of ClC-1 Chloride Channel Pre-mRNA and Hyperexcitability of Skeletal Muscle in Myotonic Dystrophy.. Molecular Cell.

[14] Seznec H, Agbulut O, Sergeant N, Savouret C, Ghestem A (2001). Mice transgenic for the human myotonic dystrophy region with expanded CTG repeats display muscular and brain abnormalities.. Hum Mol Genet.

[15] Mankodi A, Logigian E, Callahan L, McClain C, White R (2000). Myotonic dystrophy in transgenic mice expressing an expanded CUG repeat.. Science.

[16] Fardaei M, Larkin K, Brook JD, Hamshere MG (2001). In vivo co-localisation of MBNL protein with DMPK expanded-repeat transcripts.. Nucleic Acids Research.

[17] Fardaei M, Rogers MT, Thorpe HM, Larkin K, Hamshere MG (2002). Three proteins, MBNL, MBLL and MBXL, co-localize in vivo with nuclear foci of expanded-repeat transcripts in DM1 and DM2 cells.. Hum Mol Genet.

[18] de Haro M, Al-Ramahi I, De Gouyon B, Ukani L, Rosa A (2006). MBNL1 and CUGBP1 modify expanded CUG-induced toxicity in a Drosophila model of myotonic dystrophy type 1.. Hum Mol Genet.

[19] Chen W, Liang Y, Deng W, Shimizu K, Ashique AM (2003). The zinc-finger protein CNBP is required for forebrain formation in the mouse.. Development.

[20] Abe Y, Chen W, Huang W, Nishino M, Li Y-P (2006). CNBP regulates forebrain formation at organogenesis stage in chick embryos.. Developmental Biology.

[21] Weiner AM, Allende ML, Becker TS, Calcaterra NB (2007). CNBP mediates neural crest cell expansion by controlling cell proliferation and cell survival during rostral head development.. J Cell Biochem.

[22] Chen W, Wang Y, Abe Y, Cheney L, Udd B (2007). Haploinsuffciency for Znf9 in Znf9+/− Mice Is Associated with Multiorgan Abnormalities Resembling Myotonic Dystrophy.. Journal of Molecular Biology.

[23] Reddy S, Smith DB, Rich MM, Leferovich JM, Reilly P (1996). Mice lacking the myotonic dystrophy protein kinase develop a late onset progressive myopathy.. Nat Genet.

[24] Huichalaf C, Schoser B, Schneider-Gold C, Jin B, Sarkar P (2009). Reduction of the rate of protein translation in patients with myotonic dystrophy 2.. J Neurosci.

[25] Salisbury E, Schoser B, Schneider-Gold C, Wang GL, Huichalaf C (2009). Expression of RNA CCUG repeats dysregulates translation and degradation of proteins in myotonic dystrophy 2 patients.. Am J Pathol.

[26] Gerbasi VR, Link AJ (2007). The Myotonic Dystrophy Type 2 Protein ZNF9 Is Part of an ITAF Complex That Promotes Cap-independent Translation.. Mol Cell Proteomics.

[27] Michelotti EF, Tomonaga T, Krutzsch H, Levens D (1995). Cellular nucleic acid binding protein regulates the CT element of the human c-myc protooncogene.. J Biol Chem.

[28] Pellizzoni L, Lotti F, Rutjes SA, Pierandrei-Amaldi P (1998). Involvement of the Xenopus laevis Ro60 autoantigen in the alternative interaction of La and CNBP proteins with the 5′UTR of L4 ribosomal protein mRNA.. J Mol Biol.

[29] Pellizzoni L, Lotti F, Maras B, Pierandrei-Amaldi P (1997). Cellular nucleic acid binding protein binds a conserved region of the 5′ UTR of Xenopus laevis ribosomal protein mRNAs.. Journal of Molecular Biology.

[30] Cardinali B (2003). La protein is associated with terminal oligopyrimidine mrnas in actively translating polysomes.. Journal of Biological Chemistry.

[31] Sallinen R, Vihola A, Bachinski LL, Huoponen K, Haapasalo H (2004). New methods for molecular diagnosis and demonstration of the (CCTG)n mutation in myotonic dystrophy type 2 (DM2).. Neuromuscul Disord.

[32] Sundrud MS, Grill SM, Ni D, Nagata K, Alkan SS (2003). Genetic reprogramming of primary human T cells reveals functional plasticity in Th cell differentiation.. J Immunol.

[33] Fleischer TC, Weaver CM, McAfee KJ, Jennings JL, Link AJ (2006). Systematic identification and functional screens of uncharacterized proteins associated with eukaryotic ribosomal complexes.. Genes Dev.

[34] Antons AK, Wang R, Oswald-Richter K, Tseng M, Arendt CW (2008). Naive Precursors of Human Regulatory T Cells Require FoxP3 for Suppression and Are Susceptible to HIV Infection.. J Immunol.

[35] Motsinger A, Haas DW, Stanic AK, Van Kaer L, Joyce S (2002). CD1d-restricted human natural killer T cells are highly susceptible to human immunodeficiency virus 1 infection.. J Exp Med.

[36] Pyronnet S, Pradayrol L, Sonenberg N (2000). A cell cycle-dependent internal ribosome entry site.. Mol Cell.

[37] Semler B, Waterman M (2008). Ires-Mediated pathways to polysomes: nuclear versus cytoplasmic routes.. Trends in Microbiology.

[38] Spriggs KA, Bushell M, Mitchell SA, Willis AE (2005). Internal ribosome entry segment-mediated translation during apoptosis: the role of IRES-trans-acting factors.. Cell Death Differ.

[39] Stoneley M, Willis AE (2004). Cellular internal ribosome entry segments: structures, trans-acting factors and regulation of gene expression.. Oncogene.

[40] Merrick WC (1979). Purification of protein synthesis initiation factors from rabbit reticulocytes.. Methods Enzymol.

[41] Link AJ, Fleischer TC, Weaver CM, Gerbasi VR, Jennings JL (2005). Purifying protein complexes for mass spectrometry: applications to protein translation.. Methods.

[42] Link AJ, Eng J, Schieltz DM, Carmack E, Mize GJ (1999). Direct analysis of protein complexes using mass spectrometry.. Nat Biotechnol.

[43] Bagni C, Greenough WT (2005). From mrnp trafficking to spine dysmorphogenesis: the roots of fragile x syndrome.. Nature Reviews Neuroscience.

[44] Holland EC, Sonenberg N, Pandolfi PP, Thomas G (2004). Signaling control of mRNA translation in cancer pathogenesis.. Oncogene.

[45] Sonenberg N, Hinnebusch AG (2009). Regulation of translation initiation in eukaryotes: mechanisms and biological targets.. Cell.

[46] Scheper GC, Proud CG, van der Knaap MS (2006). Defective translation initiation causes vanishing of cerebral white matter.. Trends Mol Med.

[47] Yoon A, Peng G, Brandenburger Y, Zollo O, Xu W (2006). Impaired control of IRES-mediated translation in X-linked dyskeratosis congenita.. Science.

[48] Crosio C, Boyl PP, Loreni F, Pierandrei-amaldi P, Amaldi F (2000). La protein has a positive effect on the translation of TOP mRNAs in vivo.. Nucleic Acids Research.

[49] Ewing RM, Chu P, Elisma F, Li H, Taylor P (2007). Large-scale mapping of human protein-protein interactions by mass spectrometry.. Mol Syst Biol.

[50] Yuan Y, Compton SA, Sobczak K, Stenberg MG, Thornton CA (2007). Muscleblind-like 1 interacts with RNA hairpins in splicing target and pathogenic RNAs.. Nucleic Acids Research.

[51] Warf MB, Berglund JA (2007). MBNL binds similar RNA structures in the CUG repeats of myotonic dystrophy and its pre-mRNA substrate cardiac troponin T.. Rna.

